# You can bring plankton to fecal indicator organisms, but you cannot make the plankton graze: particle contribution to *E. coli* and MS2 inactivation in surface waters

**DOI:** 10.1128/msphere.00656-24

**Published:** 2024-10-03

**Authors:** Lauren C. Kennedy, Ava M. Mattis, Alexandria B. Boehm

**Affiliations:** 1 Department of Civil and Environmental Engineering, Stanford University, Stanford, California, USA; 2 Department of Civil Engineering, The University of Texas at El Paso, El Paso, Texas, USA; University of Wisconsin-Madison, Madison, Wisconsin, USA

**Keywords:** fecal indicator organism, persistence, grazer, plankton, surface water, environmental water, particle content, MS2, *Escherichia coli* K-12, pathogen pollution, fate of fecal indicator, salinity

## Abstract

Organisms that are associated with feces (“fecal indicator organisms”) are monitored to assess the potential for fecal contamination of surface water bodies in the United States. However, the effect of the complex mixtures of chemicals and the natural microbial community within surface water (“particles”) on fecal indicator organism persistence is not well characterized. We aimed to better understand how particles, including biological (e.g., potential grazers) and inert (e.g., minerals) types, affect the fecal indicator organisms *Escherichia coli* K-12 (“*E. coli*”) and bacteriophage MS2 in surface waters. A gradient of particles captured by a 0.2-µm-pore-size filter (“large particles”) was generated, and the additional particles and dissolved constituents that passed through the filter were deemed “small particles.” We measured the ratio of MS2 and *E. coli* that survived over a 24-h incubation period for each condition (0%–1,000% large-particle concentration in raw water) and completed a linear regression that included large- and small-particle coefficients. Particles were characterized by quantifying plankton, total bacterial cells, and total solids. *E. coli* and MS2 persistence was not significantly affected by large particles, but small particles had an effect in most waters. Small particles in higher-salinity waters had the largest, negative effect on *E. coli* and MS2 survival ratios: Significant small-particle coefficients ranged from −1.7 to −5.5 day^−1^ in the marine waters and −0.89 to −3.2 day^−1^ in the fresh and estuarine waters. This work will inform remediation efforts for impaired surface water bodies.

**IMPORTANCE:**

Many surface water bodies in the United States have organisms associated with fecal contamination that exceed regulatory standards and prevent safe recreation. The process to remediate impaired water bodies is complicated because these fecal indicator organisms are affected by the local environmental conditions. For example, the effect of particles in surface water on fecal indicator concentrations are difficult to quantify in a way that is comparable between studies and water bodies. We applied a method that overcomes this limitation to assess the effects of large particles, including natural plankton that could consume the seeded fecal indicator organisms. Even in environmental water samples with diverse communities of plankton present, no effect of large particles on fecal indicator concentrations was observed. These findings have implications for the interpretation and design of future studies, including that particle characterization of surface water may be necessary to assess the fate of fecal indicators.

## INTRODUCTION

In the 2017 United States National Water Quality Inventory, over 50% of the assessed rivers, bays, and estuaries were unable to support one or more of their uses, with pathogen pollution as a top cause of impairment (1). Pathogen pollution results from contamination of surface water with feces (e.g., from sewage and septic tanks) and is monitored using organisms that are associated with feces (“fecal indicator organisms”; e.g., *Escherichia coli* and *Enterococcus* spp.) (1). To remediate impaired waters, total maximum daily loads (TMDLs) are calculated for each waterbody and pollutant, including fecal indicator organisms. This process is complicated because the sources of contamination can be difficult to identify, and the effects of some of the natural processes that modulate the concentrations of fecal indicator organisms in surface water have not been well characterized (2). Unfortunately, climate change, combined with aging sewage infrastructure, is expected to exacerbate surface water contamination in the coming years (3). Exposure to contaminated waters through recreation can lead to illnesses; an estimated 90 million recreational-waterborne illnesses occur each year in the United States (4). In addition, degraded water quality can adversely affect property values adjacent to impaired waterbodies (5, 6) and individual wellbeing (7). Therefore, there is an urgent need to better understand sources, fate, and transport of pathogen pollution in surface waters.

This work focuses on better understanding how natural processes modulate concentrations of fecal indicator organisms in surface waters. Previous work has established that higher temperatures and increased sunlight exposure reduce the persistence of fecal indicator organisms in environmental waters (8–11). The effect of salinity on fecal indicator organism persistence has been equivocal with some studies showing lower, equal, or higher persistence in more saline waters compared to freshwaters (9, 11, 12). Similarly, the effect of the complex mixtures of chemicals and the natural microbial community in surface water (“particles”) on pathogen or fecal indicator organism persistence in the environment has been equivocal because studies have found them to be both protective and antagonist. For example, some plankton have been shown to consume viruses and bacteria, reducing their concentrations (13–18), while others have been shown to take up and then subsequently release viable bacteria (19). In addition, beach sand has been shown to serve as a reservoir for *E. coli* (14), but natural surface water bacteria have been shown to compete with seeded fecal indicator bacteria for nutrients, which can decrease their persistence (12, 20, 21).

A number of studies have examined the effect of particles on pathogens or fecal indicator organisms in environmental water, wherein the surface water microbial community has been controlled through filter sterilization (15, 22–24), autoclaving (15, 25, 26), and/or addition of eukaryotic inhibitors (20, 22, 27). Separating the effect of the surface water microbial community, including bacteria and protozoa (“biological particles”), from that of other impurities in the surface water, including soil and sand (“inert particles”), is difficult. Multiple studies have found longer persistence of MS2 and *E. coli* in filter-sterilized surface water, in autoclaved surface water, or in surface water that has been seeded with eukaryotic inhibitors compared to raw surface water (10, 12, 20, 23–25, 28–33), but others have found no significant effect of biological and inert particles on the persistence of *E. coli* in filtered compared to raw surface waters (34, 35). The differences between findings could be explained by the complex interactions between natural particles in surface water and seeded microorganisms (36–38) or differences in methods that make these studies difficult to compare. For example, autoclaving can affect the inert particles present (e.g., dissolved organic carbon [39]), and eukaryotic inhibitors may not inhibit all biological particles (40). Landry and Hassett (13) developed an approach that allows one to quantify the effect of biological and inert particles on pathogen persistence by comparing their survival ratios in dilutions of raw water with filter-sterilized water. This method has been applied to study enterococci in two coastal environmental water samples (41), but to our knowledge, this method has not been applied to enteric viruses or other seeded bacteria.

We aimed to quantify and compare the effect of particles on the persistence of fecal indicator organisms in environmental surface waters and on different types of microorganisms (i.e., viruses compared to bacteria). Two fecal indicator organisms were included in this study: (i) *E. coli* K-12 was included because it is commonly used to monitor fecal contamination in surface water, and (ii) bacteriophage MS2 was included because F+ coliphages, like MS2, are present at high concentrations in wastewater, and they are persistent in environmental waters (42). This work will inform surface water remediation efforts and contribute to the parameterization of fecal indicator organism persistence in surface waters.

## MATERIALS AND METHODS

### Raw water collection

Water was collected from five locations on six dates (one location was sampled twice; Table 1). For each sampling event, four to six, 5.4-L samples were collected in sterile bags (Whirl-pak, Nasco Sampling, WI, USA) by gently filling the bag on its side so as not to harm any plankton potentially present in the sample; multiple samples were collected at each site owing to logistical constraints associated with site accessibility. The first 5.4-L sample collected was used to measure the temperature and salinity onsite as well as for the experiments and most measurements conducted in this study. The subsequent samples were used for plankton enumeration only. The bags were transported on ice and kept at 4°C until use later that day (T0). The salinity, temperature, turbidity, pH, and UVvis absorbance were measured as described previously (23, 43). In addition, plankton in the unaltered environmental water (“raw water”) were concentrated onto 70-µm opening mesh, preserved with 70% ethanol, stained with 0.04% rose bengal, and enumerated by microscopy following previous methods (41). Total bacterial cell count was assessed with 4′,6-diamidino-2-phenylindole (DAPI) stain following methods described previously with slight modifications (44). Briefly, bacteria previously fixed using 2% paraformaldehyde were vacuum filtered onto a 0.2-µm-pore-size polycarbonate filter (MilliporeSigma, Burlington, MA, USA), stained with Vectashied with DAPI (Fisher, Waltham, MA, USA), imaged with an in epifluorescence microscope (Nikon, Minato City, Tokyo, Japan), and enumerated using ImageJ (v1.53t). Total solids were quantified following Standard Methods 2540 by evaporating water from a raw water sample in a drying oven and quantifying the dried mass. Total solids represent total dissolved solids and total suspended solids. Additional information is in the SI.

**TABLE 1:** Water was collected from five locations on six dates^*a*^

Water	Sampling date (T0)	Location	Coordinates
San Gregorio State Beach	4/19/23	San Gregorio, CA, USA	37.322 N, −122.404 W
San Pedro Beach—April	4/26/23	Pacifica, CA, USA	37.598 N, −122.503 W
San Pedro Creek	5/24/23	Pacifica, CA, USA	37.5962 N, −122.5055 W
San Francisco Bay	5/31/23	Palo Alto, CA, USA	37.4578 N, −122.1010 W
Lake Chabot	6/7/23	Castro Valley, CA, USA	37.7175 N, −122.1045 W
San Pedro Beach—June	6/13/23	Pacifica, CA, USA	37.598 N, −122.503 W

^*a*^ The first column includes the name used herein, followed by the sample date that the water was collected and day 0 of the corresponding experiment (T0) (given as month/day/year), the location of the water body, and the coordinates of the sampling site.

### *E. coli* and MS2 inoculant preparation

Bacteriophage MS2 (American Type Culture Collection [ATCC] 15597-B1) and *E. coli* K-12 (ATCC 29425) glycerol stock preparation and experimental preparation are detailed in the SI. Log-phase *E. coli* was centrifuged at 9,500 × *g* for 5 min. The pellet was washed three times in autoclave-sterilized phosphate-buffered saline (PBS; Thermo Fisher Scientific, MA, USA) and then resuspended such that the concentration was estimated based on the absorbance of the log-phase *E. coli* to be ~1.4 × 10^8^ colonies/mL (“*E. coli* inoculant”). For MS2, a glycerol stock was thawed and serially diluted to be ~1.4 × 10^8^ plaque-forming units (PFU)/mL in autoclave-sterilized PBS (“MS2 inoculant”). MS2 glycerol stocks were thawed once. *E. coli* and MS2 inoculants were kept on ice until use.

### Experimental setup

**Fig 1:**
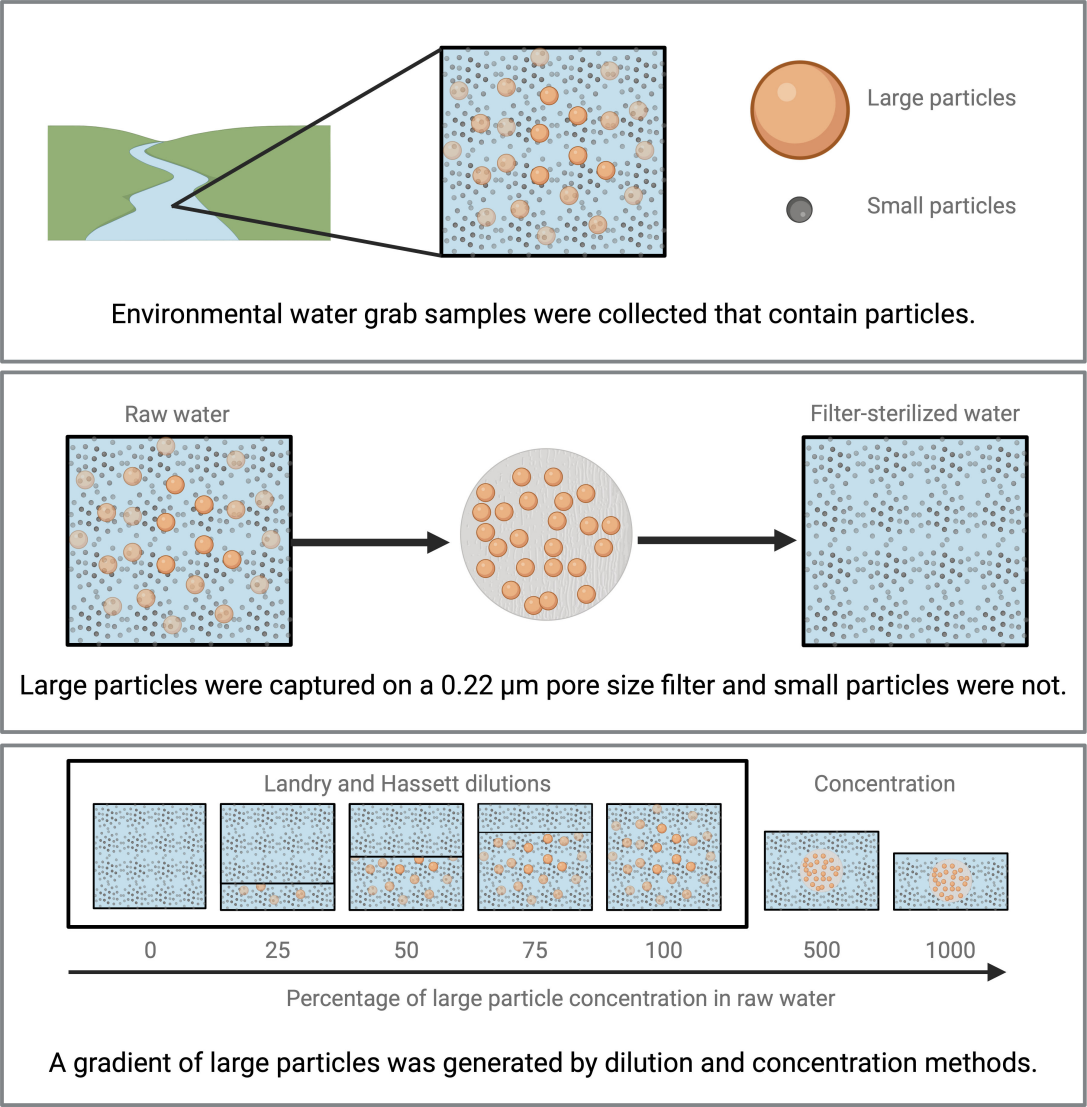
Environmental water grab samples were collected of “raw water” (top). The raw water was filtered using a 0.22-µm-pore-size filter, and particles that passed through the filter were deemed “small particles,” while particles retained on the filter were deemed “large particles” (e.g., bacteria, protists, sand, etc.; middle). To generate a gradient of large particles, raw water was mixed with different proportions of filter-sterilized water (“dilution”), and raw water was filtered onto a membrane filter that was resuspended in filter-sterilized water (“concentration”; bottom). MS2 and *E. coli* were seeded into each percentage in the large particle gradient, and their 24-h log survival ratios were compared (equation 1) (created with Biorender.com).

The log survival ratio of *E. coli* and MS2 was assessed for different concentrations of the particles captured on a 0.22-µm-pore-size filter (“large particles;” Fig. 1) from surface water. The particles and dissolved constituents that passed through the filter include small particles, such as some colloidal particles, viruses, and phage, as well as dissolved constituents (deemed “small particles” hereafter). To produce this gradient of large particles, two techniques were applied. The first technique was to dilute the raw water with water containing only small particles (“filter-sterilized water”). Five conditions were compared: 0%, 25%, 50%, 75%, and 100% of large particles in raw water (“Landry and Hassett dilutions”) (13). The second approach was to capture large particles on a filter and resuspend them in filter-sterilized water (“concentrated raw water”). ~250 and ~500 mL of raw water were vacuum filtered onto a 0.22-μm-pore-size polyethersulfone membrane filter (Millipore Express PLUS, MA, USA). The filters were resuspended, following Olive et al. (15) with slight modifications, in 50 mL of the filtrate and oscillated at 4°C for 30 min (i.e., to produce 500% and 1,000% large-particle concentration in raw water). Filtration has been applied to concentrate and enumerate viable bacteria (e.g., through the United States Environmental Protection Agency [USEPA] method 1603) and protists (15) previously. These approaches were used in three experiments comparing the log survival ratio of *E. coli* and MS2 (i) after 24 and then 48 h of incubation in one environmental water (“incubation time experiment”), (ii) in two environmental waters applying Landry and Hassett dilutions (13) (“dilution-only experiment”), and (iii) in four environmental waters adapting the methods of Landry and Hassett to include two concentration conditions (“dilution and concentration experiment”). The experiments were conducted at a common coastal average surface water temperature (15°C [45]) and in dark conditions to reduce confounding effects from light.

For all of the environmental water samples collected, 21 15-mL sterile centrifuge tubes covered in tin foil served as reactors, which included an experimental negative control, duplicate reactors for each of the five conditions, and separate reactors for MS2 (10 reactors) and *E. coli* (10 reactors). The duplicate reactors for each condition were derived from the same grab sample. One reactor consisted of autoclave-sterilized PBS (“negative control reactor”) during each set of experiments. MS2 and log-phase *E. coli* were seeded into separate, duplicate, reactors for each condition at initial concentrations of 4.0 × 10^6^ ± 2.6 × 10^6^ PFU/mL and 4.4 × 10^5^ ± 3.8 × 10^5^ CFU/mL respectively (where error is the standard deviation). The final reactor volume was 10 mL, and the reactors were stored at 15°C in the dark oscillating at 100 revolutions per minute. *E. coli* and MS2 were quantified immediately after inoculation and mixing (T0) and after 24 h of incubation (T1). For one environmental water sample (incubation time experiment only), *E. coli* and MS2 were also quantified after 2 days (T2) of incubation for comparison; additional days were not considered due to concerns about bottle effects. One milliliter of sample was collected at each time point and was kept on ice until quantification.

### Quantification o*f E. coli* and MS2

Samples collected at each time point were quantified immediately following collection and serial dilution in autoclave-sterilized PBS (Thermo Fisher Scientific, MA, USA). MS2 was quantified using the double agar layer plaque assay in USEPA method 1602 with log-phase F_amp_ and tryptic soy broth (TSB) with 15 mg/L of streptomycin and ampicillin, as described previously (23). *E. coli* was quantified following USEPA method 1603 on membrane Thermotolerant *E. coli* (mTEC) agar (Becton Dickenson, NJ, USA). However, the spread plate method was used for quantification, and incubation was completed at the ATCC recommended temperature for *E. coli* 29425 (37°C). Additional details are in the SI.

Quality assurance and control were completed. First, background concentrations of *E. coli* or F+ coliphage in the environmental water samples were assessed: (i) six uninoculated raw water samples; (ii) four uninoculated 1,000% concentrated samples; and (iii) six uninoculated filter-sterilized water samples that were assessed with T0 samples. Additional experimental and technical controls were included; (iv) 12 technical blanks, which consisted of TSB with antibiotics for MS2 and TSB without antibiotics for *E. coli* were quantified at each time point; (v) six technical negative controls, which consisted of *Enterococcus faecalis* (*E. faecalis*) spread onto mTEC media that were assessed to ensure selectivity of the media (i.e., verifying that no colonies of *E. faecalis* grew on a media that is selective for *E. coli*), (vi) six “viability” controls, which consisted of *E. faecalis* spread on nutrient agar that were assessed to ensure selectivity of the media (i.e., verifying that *E. faecalis* was viable when serving as a technical negative control described in part v); (vii) six technical positive controls of diluted glycerol stocks for both *E. coli* and MS2 to assess the sensitivity of the respective media; and (viii) six experimental negative controls, which consisted of reactors filled with PBS. Finally, reactors for each microorganism and environmental water condition were completed in duplicate.

### Data analysis

Data analysis was completed in R (v4.1.3). Linear regressions of the log survival ratio [ln(*N*(*t*)/*N*_0_)] assessed over an incubation time period (*t*) were applied for each experiment and microorganism (equation 1), where “*N*(*t*)” is the concentration of MS2 or *E. coli* over time, *t*, and “*N*_0_” is the concentration of MS2 or *E. coli* at the beginning of the experiment “T0” (Table 1), following Landry and Hassett (13). “Large particle” and “small particle” decay coefficients (“β_large particle_” and “β_small particle_,” respectively) were assessed by generating a gradient of large-particle concentrations as measured by the percentage of large-particle concentration in raw water (“*p*”). The lm() function in R was used to calculate the slope (β_large particle_) and the intercept (β_small particle_) as well as their standard errors, and *P* values.


(1) $$\frac{1}{t}\ln\left(\frac{N(t)}{N_{0}}\right)=\beta_{\text{small particle }}+\beta_{\text{large particle }}*p+\varepsilon$$

Multiple linear regressions were used for three experiments: the incubation time experiment (equation 2), the dilution-only experiment (equation 3), and the dilution and concentration experiment (equation 4). For the incubation time experiment, a multiple linear regression was used to assess the effect of incubation time (“*T*”) on the persistence of MS2 and *E. coli* (equation 2). A dummy variable for 48 h of incubation (“*T*”) was compared to the reference condition, 24 h of incubation. For the dilution-only experiment, a multiple linear regression was used to compare the log survival ratio of *E. coli* and MS2 assessed after 24 h of incubation for two environmental waters. SG was a dummy variable for San Gregorio State Beach water, and the reference condition was San Pedro Beach—April water, the first of the two water samples to be collected. For the dilution and concentration experiment, a multiple linear regression was used to compare the log survival ratio of *E. coli* and MS2 assessed after 24 h of incubation for four environmental waters. The reference condition was the first of the four water samples to be collected, Lake Chabot, which was compared using dummy variables for San Francisco Bay (“SF”), San Pedro Beach—June (“SPB”), and San Pedro Creek (“SPC”). The lm() function in *R* was used to determine all coefficients, their standard errors, and their *P* values. Additional details are in the SI.


(2) $$\frac{1}{t}\ln\left(\frac{N(t)}{N_{0}}\right)=\beta_{0}+\beta_{1}p+\beta_{2}T+\beta_{12}pT+\varepsilon$$

(3) $$\frac{1}{t}\ln\left(\frac{N(t)}{N_{0}}\right)=\beta_{0}+\beta_{1}p+\beta_{2}SG+\beta_{12}pSG+\varepsilon$$

(4) $$\frac{1}{t}\ln\left(\frac{N(t)}{N_{0}}\right)=\beta_{0}+\beta_{1}p+\beta_{2}SF+\beta_{12}pSF+\beta_{3}SPC+\beta_{13}pSPC+\beta_{4}SPB+\beta_{14}pSPB+\varepsilon$$

## RESULTS

### Quality assurance and quality control

We measured *E. coli* and F+ coliphage in the environmental water samples, and no colonies or plaques were detected in the raw water (100% large particles) or concentrated raw water (1,000% large particles). We included technical blanks, technical negative controls, technical positive controls, and experimental negative controls. Technical blanks had no colonies or plaques of *E. coli* and MS2, respectively. For *E. coli* technical negative controls, we first checked that *E. faecalis* was viable on nutrient agar, and all six plates were positive. Then, we cultured *E. faecalis* on mTEC agar, and all six plates were negative. The six technical positive controls that were positive and had an average of 7.7 × 10^7^ ± 2.9 × 10^7^ CFU/mL of *E. coli* and 4.5 × 10^11^ ± 2.4 × 10^11^ PFU/mL of MS2. No colonies or plaques were detected on 25 out of the 26 plates for the negative control reactors. For one time point, one colony of *E. coli* was detected in the negative control reactor, but the concentration was ~93% lower than the lowest concentration of *E. coli* in the reactors with environmental water at any raw water percentage 0%–1,000% (50 CFU/mL compared to 745 CFU/mL respectively).

### Incubation time experiment: 24 compared to 48 h

First, we compared the log survival ratio of *E. coli* and MS2 after 24 and then 48 h of incubation. For this experiment, a gradient of large-particle concentrations was generated by diluting raw water with filter-sterilized water to produce reactors with five percentages of large particles in raw water, from 0% to 100% (Fig. 1). In this experiment, no significant effect of large particles on the log survival ratio of *E. coli* or MS2 was observed after 24 or after 48 h. However, small particles had a significant, negative effect on the log survival ratio of *E. coli* and MS2 after 24 and after 48 h (β_small particle_ ranging from −1.7 to −2.9 day^−1^ *P* < 0.01; Table S1; Fig. S1). To assess whether the incubation time influenced the effect of particles on the log survival ratio of microorganisms, multiple linear regressions were applied (equation 2). The effects of large and small particles on the log survival ratio of MS2 were similar after 24 compared to 48 h of incubation (Table S2, *F*-test *P*-value = 0.22). For *E. coli*, small particles reduced the log survival ratio significantly more when the survival ratio was assessed after 24 h compared to when the survival ratio was assessed after 48 h of incubation (β_0_ = −3.0 day^−1^ *P*-value < 0.0001 compared to β_2_ = 0.64 day^−1^ *P*-value < 0.01; Table S3). However, 24 h was set as the incubation time for subsequent experiments because the effects of large particles on the log survival ratio of MS2 and *E. coli* were similar after 24 or 48 h.

### Dilution-only experiments (0%–100% of large-particle concentration in raw water)

The log survival ratio of *E. coli* and MS2 was assessed after 24 h of incubation (“24-h log survival ratio”) for two environmental waters: San Gregorio State Beach and San Pedro Beach—April. For these experiments, a gradient of large-particle concentrations was generated by diluting raw water with filter-sterilized water to produce reactors with five percentages of large particles in raw water, from 0% to 100% (Fig. 1). Large particles did not significantly affect the 24-h log survival ratio of either microorganism in either environmental water. However, small particles significantly reduced the 24-h log survival ratio of *E. coli* and MS2 in water from San Gregorio State Beach and from San Pedro Beach—April (β_small particle_ ranging from −2.3 to −5.5 day^−1^ *P* < 0.01; Table S1; Fig. S2). To assess whether the unique biological and inert particles in each water affected the 24-h log survival ratio of MS2 and *E. coli*, multiple linear regressions were applied (equation 3). The effects of large and small particles from both waters on the 24-h log survival ratio of *E. coli* were similar (Table S4, *F*-test *P*-value = 0.080). However, small particles from San Pedro Beach—April reduced the 24-h log survival ratio of MS2 significantly more compared to those from San Gregorio State Beach (β_2_ = −2.6 day^−1^ *P* < 0.01 compared to β_0_ = −2.9 day^−1^ *P* <0.001; Table S5).

### Dilution and concentration experiments (0%–1000% of large-particle concentration in raw water)

The 24-h log survival ratio of *E. coli* and MS2 was assessed for two marine waters and two fresh waters. Similar to the dilution-only experiments, a gradient of large-particle concentrations was generated by diluting raw water with filter-sterilized water. However, in these experiments, the Landry and Hassett dilutions were supplemented with higher concentrations of large particles that were generated by capturing particles on a filter and resuspending them in filter-sterilized water. In these experiments, reactors with seven percentages of large particles in raw water, from 0% to 1,000%, were compared (Fig. 1).

**Fig 2:**
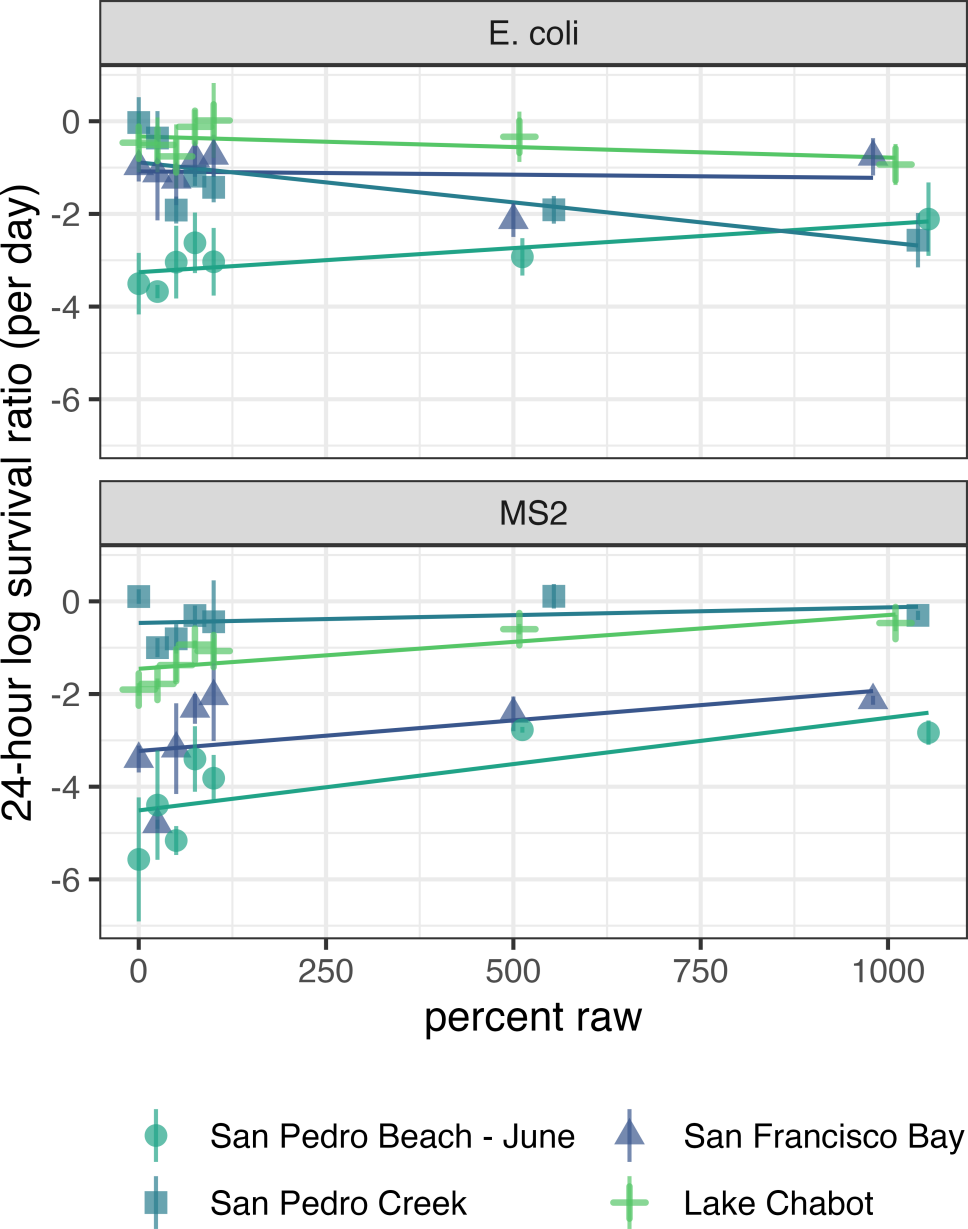
Water was collected from four locations (distinguished by color). Dilutions with filter-sterilized water consisted of 0%, 25%, 50%, 75%, and 100% large-particle concentration in raw water (*x*-axis), and concentrations of raw water were prepared by membrane filtration and resuspending particles in the filtrate to achieve ~500% and 1,000% large-particle concentration in raw water. The apparent rate of change (day^−1^) was assessed after 24 h incubating in the dark at 15°C for *E. coli* (top) and MS2 (bottom). The error bars denote the standard deviation of duplicate reactors with water from the same grab sample. The coefficients were calculated using equation 1.

The effects of large and small particles on the 24-h log survival ratio of *E. coli* differed between environmental waters (Table S1; Fig. 2). Particles from Lake Chabot did not affect the 24-h log survival ratio of *E. coli*, while large and small particles from San Pedro Beach—June significantly affected the 24-h log survival ratio of *E. coli*. Large particles from San Pedro Beach—June significantly raised the 24-h log survival ratio of *E. coli* (β_large particle_ = 0.0010 day^−1^ *P* < 0.05; Table S1; Fig. 2), while small particles significantly reduced the 24-h log survival ratio of *E. coli* (β_small particle_ = −3.3 day^−1^ *P* < 0.00001; Table S1; Fig. 2). Small particles from San Francisco Bay and San Pedro Creek significantly reduced the 24h log survival ratio of *E. coli* (β_small particle_ = −1.1 and −0.89 day^−1^ *P* < 0.05 respectively; Table S1; Fig. 2), but large particles from San Francisco Bay and San Pedro Creek did not have a significant effect on the 24-h log survival ratio of *E. coli*. To assess whether the unique particles in each water affected the 24-h log survival ratio of *E. coli*, multiple linear regressions were applied (equation 4). Small particles from San Francisco Bay and San Pedro Beach—June reduced the 24 h log survival ratio of *E. coli* significantly more compared to those from Lake Chabot and San Pedro Creek (β_2_ = −0.75 and β_4_ = −2.9 day^−1^ *P* <0.05; compared to β_0_ = −0.33 and β_3_ = −0.56 day^−1^ *P* > 0.05; Table S6).

Similarly, the effects of large and small particles on the 24h log survival ratio of MS2 differed between environmental waters (Table S1; Fig. 2). Particles from San Pedro Creek did not affect the 24-h log survival ratio of MS2, while large and small particles from Lake Chabot significantly affected the 24-h log survival ratio of MS2. Large particles from Lake Chabot significantly raised the 24-h log survival ratio of MS2 (β_large particle_ = 0.0012 day^−1^ *P* < 0.05; Table S1; Fig. 2), while small particles significantly reduced the 24-h log survival ratio of MS2 (β_small particle_ = −1.5 day^−1^ *P* < 0.001; Table S1; Fig. 2). Small particles from San Francisco Bay and San Pedro Beach—June significantly reduced the 24-h log survival ratio of MS2 (β_small particle_ = −3.2 and −4.5 day^−1^ *P* < 0.001 respectively), but large particles from San Francisco Bay and San Pedro Beach—June did not have a significant effect on the 24-h log survival ratio of MS2. To assess whether the unique particles in each water affected the 24-h log survival ratio of MS2, multiple linear regressions were applied (equation 4). Small particles from San Francisco Bay, San Pedro Beach—June, and Lake Chabot reduced the 24-h log survival ratio of MS2 significantly more compared to those from San Pedro Creek (β_2_ = −1.8, β_4_ = −3.1, and β_0_ = −1.5 day^−1^ *P* <0.001; compared to β_3_ = 0.99 day^−1^ *P* <0.05; Table S7).

### Biological and inert particle characterization

Physicochemical water quality characteristics were assessed for each environmental water sample (Table S8). Upon collection, all water samples ranged in temperature from 11.2°C to 20.6°C, and the salinity ranged from 0.2 to 32.7 parts per thousand (ppt). The absorbance spectra for the raw waters was highest in San Pedro Beach—June and Lake Chabot and lowest in San Pedro Beach—April and San Gregorio State Beach (Fig. S3). San Francisco Bay, San Pedro Creek, and Lake Chabot had visibly lower absorbance spectra following filtration with a 0.22-µm-pore-size filter. The turbidity of the raw water ranged from 5.4 to 14.8 nephloid turbidity units (NTU), and the pH ranged from 7.78 to 8.08.

**Fig 3:**
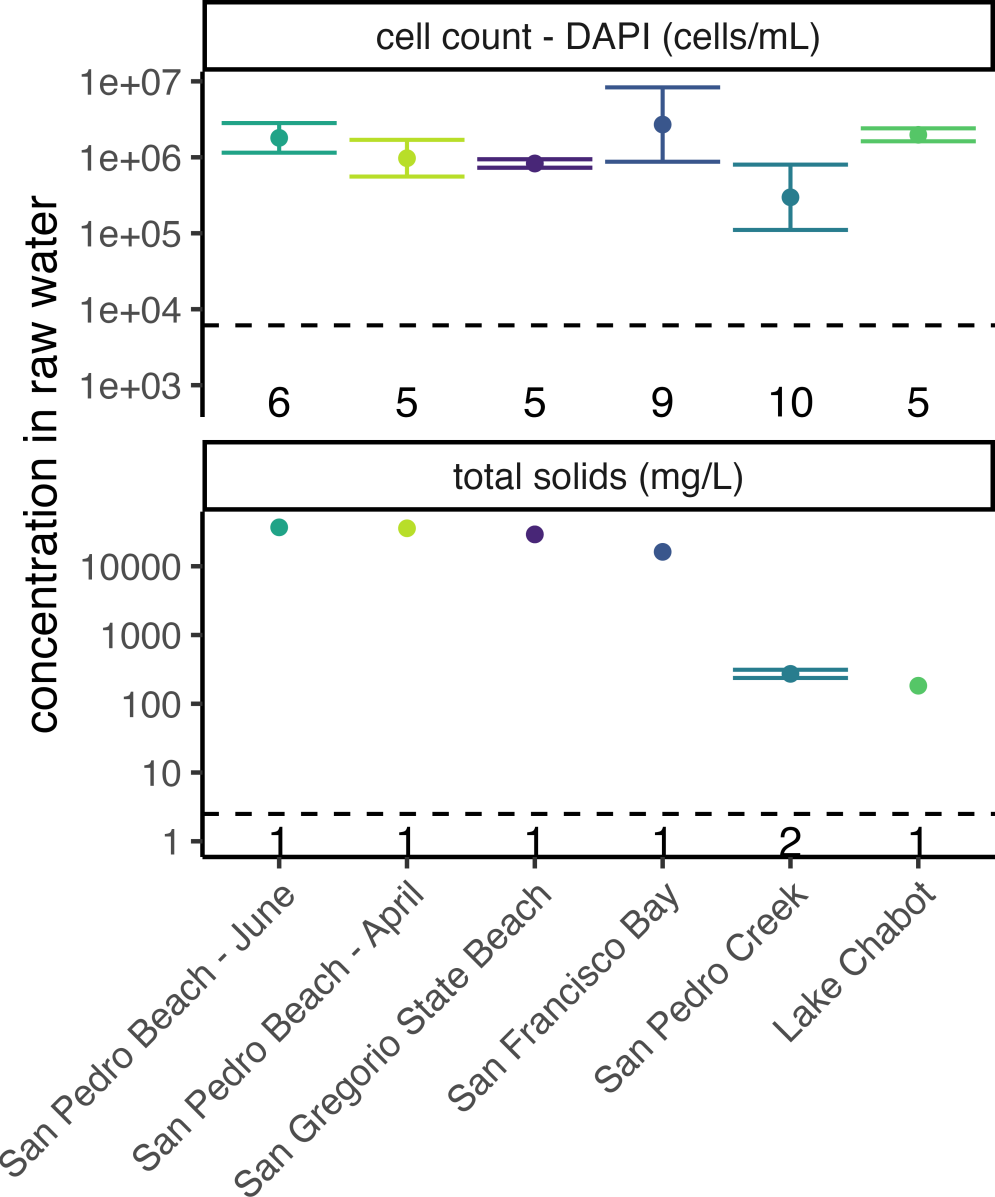
Concentrations of DAPI-stained cells (top) and total solids (bottom) in each environmental water sample included in this study (indicated by color). Error bars represent the geometric standard deviation of replicate areas counted on the same filter (top) or of triplicates (middle). The number of replicates included in the calculation of geometric mean and standard deviation plotted immediately above the *x*-axis. The dashed horizontal lines denote the detection limits for this study.

Inert and biological particles were characterized by determining bacterial cell count (i.e., through DAPI staining) and measuring total solids in the raw water. The bacterial cell counts of all waters ranged from 3.0 × 10^5^ to 2.7 × 10^6^ cells/mL, with an average of 1.3 × 10^6^ ± 8.4 × 10^5^ cells/mL (Fig. 3; where the error is the standard deviation). In addition, the total solids in the waters ranged from 230 to 3.7 × 10^4^ mg/L, where the freshwaters had an average of 230 ± 63 mg/L, and the marine waters had an average of 2.9 × 10^4^ ± 8.2 × 10^3^ mg/L (Fig. 3; where the error is the standard deviation).

**Fig 4:**
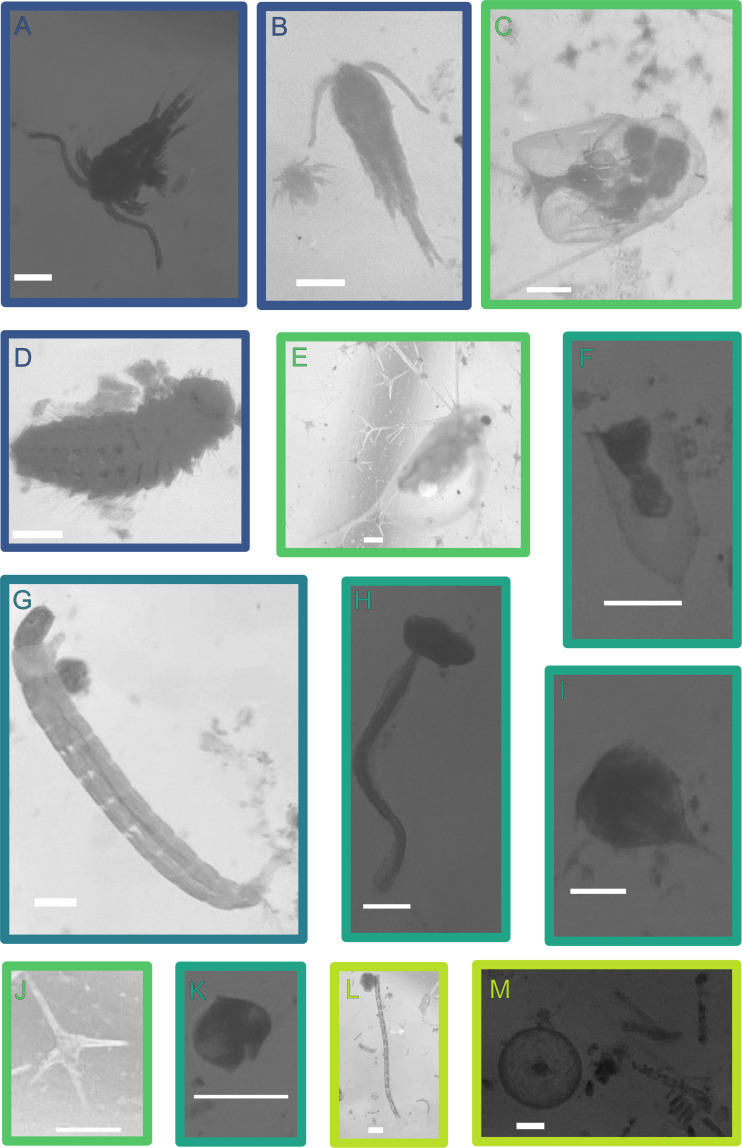
Example identified plankton where the scale bar indicates 100 µm: (**A**) adult Copepoda from San Francisco Bay, (**B**) juvenile and adult Copepoda from San Francisco Bay, (**C**) rotifera from Lake Chabot, (**D**) worm larvae from San Francisco Bay, (**E**) Cladocera from Lake Chabot, (**F**) cillate from San Pedro Beach—June, (**G**) Diptera larvae from San Pedro Creek, (**H**) Appendicularia larvae from San Pedro Beach—June, (**I**) cirripede nauplius from San Pedro Beach—June, (**J**) dinoflagellate from Lake Chabot, (**K**) dinoflagellate cyst from San Pedro Beach—June, (**L**) filamentous algae from San Pedro Beach—April, and (**M**) diatom from San Pedro Beach—April.

**Fig 5:**
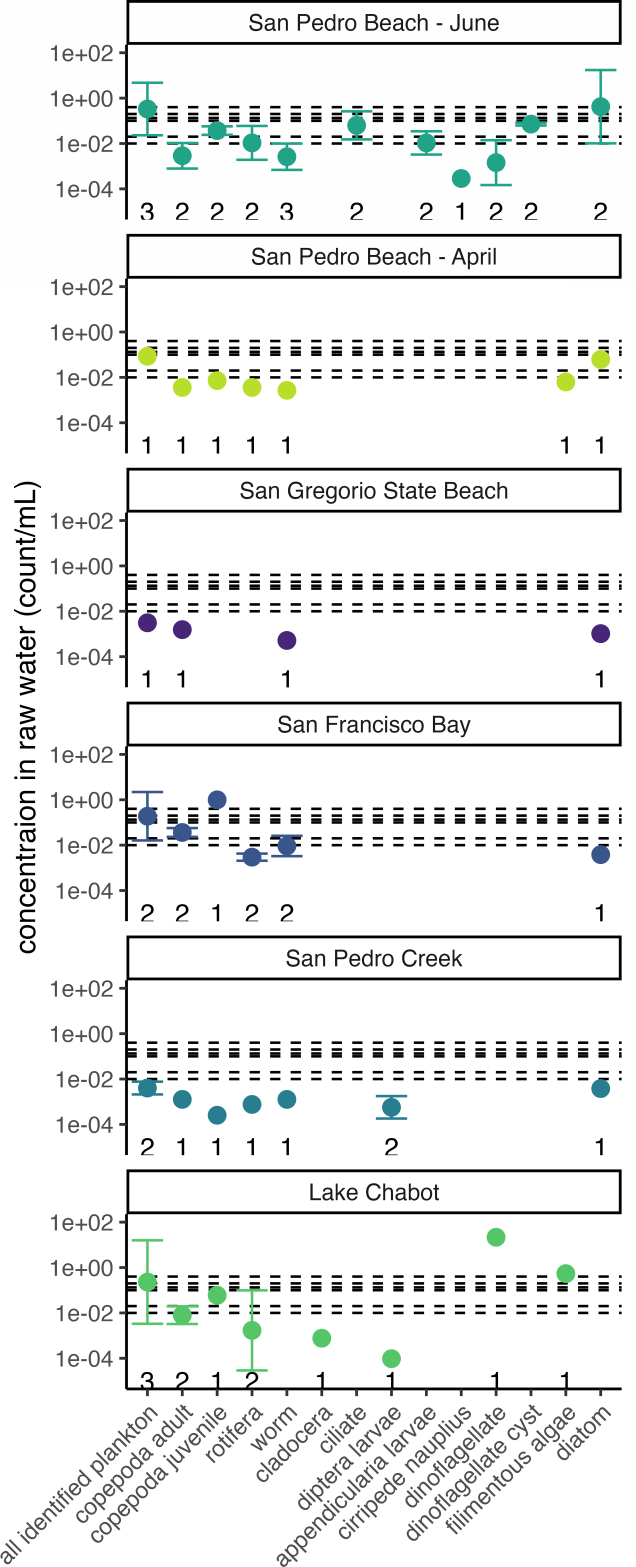
Concentration of plankton identified (*x-*axis) in each environmental water sample included in this study (indicated by color). Horizontal dashed lines denote the concentrations required to observe one organism per reactor for each condition (i.e., reactors consisting of 25%, 50%, 75%, 100%, 500%, and 1,000% of large particles in the corresponding raw water). Error bars are the geometric standard deviation from organisms that were enumerated at multiple dilutions of the rose bengal-stained, ethanol-preserved samples. The number of dilutions included in the calculation of the geometric mean and standard deviation is plotted immediately above the *x*-axis.

Biological particles were also characterized by enumerating small plankton (~50 µm to ~2 mm). Thirteen types of plankton were identified in the environmental water samples: adult Copepoda, juvenile Copepoda, Rotifera, worm larvae, Cladocera, ciliates, Diptera larvae, Appendicularia larvae, cirripede nauplii, dinoflagellates, dinoflagellate cysts, filamentous algae, and diatoms (Fig. 4). Three environmental waters included plankton at high enough concentrations to have at least one organism in the 25% large particle concentration in raw water reactors (at least 0.4 plankton per mL of raw water): 0.99 juvenile Copepoda per mL of San Francisco Bay, 22 dinoflagellates per mL and 0.54 filamentous algae per mL of Lake Chabot, and 0.42 diatoms per mL in San Pedro Beach—June (Fig. 5).

**Fig 6:**
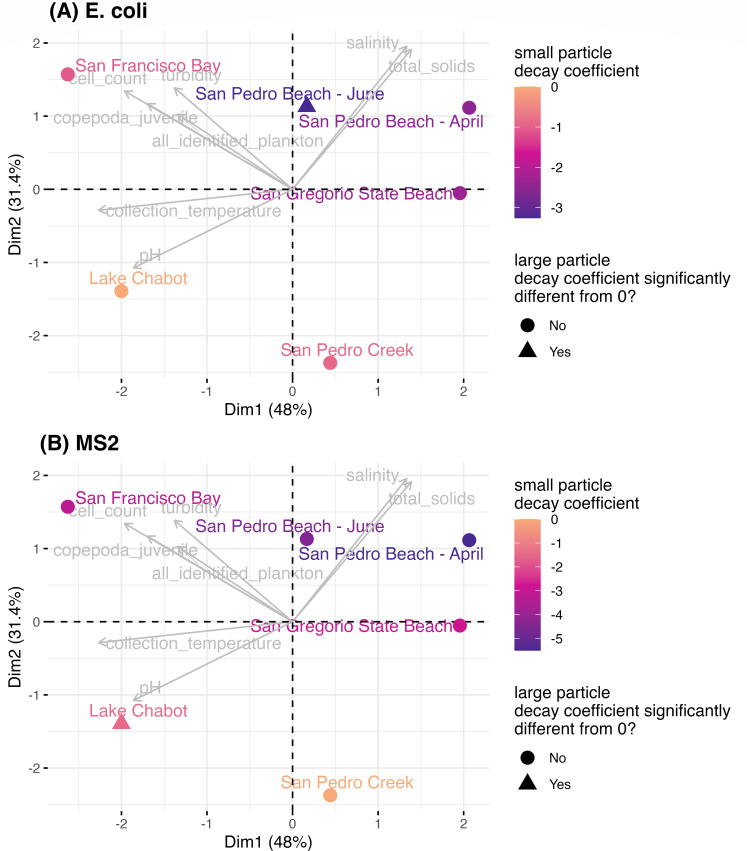
PCA biplot of the total plankton concentration, juvenile Copepoda concentration, total solids, salinity, cell count, turbidity, and pH for each raw environmental water sample in this study, with loadings shown in gray. The colors denote the small particle decay coefficients of (**A**) *E. coli* and (**B**) MS2, and the shape indicates whether the large particle decay coefficient is significantly different from 0 day^−1^.

To explore differences in biological and inert particles in the environmental water samples, principal components analysis (PCA) was completed with scaled data from total plankton concentration, juvenile Copepoda concentration, total solids, salinity, cell count, turbidity, and pH for each raw environmental water sample in this study. We included total plankton and juvenile Copepoda in the PCA because, for at least half the waters, they were present at high-enough concentrations to have at least one plankton present in the 500% reactor (>0.02 plankton per mL). The large and small particle decay coefficients that were significantly different from 0 day^−1^ for *E. coli* and MS2 were distinguished with color (Fig. 6). Of the variation between the sites, 48% could be explained by principal component 1, for which collection temperature and cell count had the highest loading. Of the variation between the sites, 31% could be explained by principal component 2, for which salinity and total solids had the highest loading.

## DISCUSSION

Despite differences in the natural planktonic communities of the environmental waters compared in this study, the effect of large particles on seeded microorganism persistence was small or negligible. Zooplankton are known to consume phytoplankton, but they also consume other zooplankton (46, 47). For example, copepods have been shown to feed on bacteria, algae, ciliates, and rotifers, and many species of copepods are cannibalistic (48). Thus, the effect of grazers on introduced microorganisms are likely dependent on the concentrations and types of plankton that could consume introduced microorganisms and the alternate food sources available. In addition, we did not observe negative effects of competition from the natural surface water bacteria on *E. coli* persistence. Previous research found that *E. coli* survival was affected by competition with natural bacteria more than that of *E. faecalis* in surface water (21), and the natural microbial community was found to reduce the persistence of seeded *E. coli* more in bulk water compared to sediments (12). Suspended sediment particles could have obscured the impact of competition on *E. coli* persistence in this study.

For two of the environmental waters in this study, a small increase in the 24-h log survival ratio of MS2 or *E. coli* was observed (a “protective effect”) in the presence of higher concentrations of large particles, including potential grazers and inert particles. Some plankton have been shown to serve as hosts for viruses and bacteria (19, 47). Indeed, Cesare et al. (19) recently found that *E. coli* that were taken up by the planktonic Cladocera, *Daphina* sp., were viable upon release. It is possible that while associated with plankton, microorganisms could be protected from inactivation by other impurities in the water. A similar effect has ben observed in drinking water distribution systems: some opportunistic pathogens can survive phagocytosis by free living amoeba during which they can be protected from chemical disinfectants like free chlorine (49). In this study, we identified Cladocera in Lake Chabot water at a low concentration (7.7 × 10^−4^ per mL), the water for which we observed a small protective effect for MS2. Furthermore, some inert particles would be classified as “large” if present in the environmental waters in this study (e.g., sand or soil) and could affect microorganism persistence. For example, researchers have found that *E. coli* persisted longer in sediment samples compared to water samples (12, 31), which suggests that inert particle content could contribute to protection of microorganisms from predation or other stressors that might cause inactivation.

For fresh compared to marine waters, no pattern in the effect of large particles on microorganism log survival ratios was observed, but small particles and water quality characteristics in most of the environmental waters included in this study contributed to the significantly greater decay of MS2 and *E. coli*. Eight measures of biological and inert particles or general water quality characteristics (i.e., salinity) of the raw water samples were evaluated using PCA. In general, the environmental waters for which small particles had the largest, negative effect on *E. coli* and MS2 24-h log survival ratios had higher salinity, higher total solid concentration, lower collection temperatures, and lower cell counts (which suggests lower nutrients; quadrant 1; Fig. 6A and B). These findings suggest that salinity contributed to the decay of MS2 and *E. coli* in the environmental waters tested.

Our findings have implications for quantifying fecal indicator organism TMDLs in impaired water bodies. Though potential grazers were present in the environmental waters in this study, they had small or negligible effects on the log survival ratios of MS2 and *E. coli*. Based on this finding, enhanced particle characterization in environmental water bodies, including assessing the potential predators present, would be necessary to parametrize pathogen and fecal indicator organism persistence in the presence of potential grazers. We also found that salinity could modulate fecal indicator concentrations in environmental waters, with significant β_small particle_ ranging from −1.7 to −5.5 day^−1^ in the waters with salinities greater than 20 ppt and −0.89 to −3.2 day^−1^ in the waters with salinities less than 20 ppt. Based on this finding, the effect of salinity on fecal indicator TMDLs in surface waters should be considered in future assessments of impaired water bodies.

## Data Availability

Data analysis code and data set are available through the Stanford Digital Repository (https://doi.org/10.25740/jn889xr4233).

## SUPPLEMENTAL MATERIAL

Supplemental Material msphere.00656-24-s0001.pdf Supplemental text, figures, and tables.

ASM does not own the copyrights to Supplemental Material that may be linked to, or accessed through, an article. The authors have granted ASM a non-exclusive, world-wide license to publish the Supplemental Material files. Please contact the corresponding author directly for reuse.
