# Supplementary material for: You can bring plankton to fecal indicator organisms, but you cannot make the plankton graze: particle contribution to E. coli and MS2 inactivation in surface waters
Source: mSphere. 2024 Oct 3;9(10):e00656-24. doi: 10.1128/msphere.00656-24 (PMC11520309; doi:10.1128/msphere.00656-24)
Supplement: Supplemental Material — Supplemental text, figures, and tables. [file msphere.00656-24-s0001.pdf]

Supplemental Material

You can bring plankton to fecal indicator organisms, but you cannot make the plankton graze: particle contribution to E. coli and MS2 inactivation in surface waters

Lauren C. Kennedy[a]*#, Ava M. Mattis[a], and Alexandria B. Boehm[a]

a. Department of Civil and Environmental Engineering, Stanford University, Stanford, California, USA

#Address correspondence to Lauren C. Kennedy lckennedy@utep.edu

*Present address: Lauren C. Kennedy, Department of Civil Engineering, University of Texas at El Paso, El Paso, Texas, USA

Supplemental Methods **2**

Glycerol stock preparation 2

E. coli, Famp, and E. faecalis experimental preparation 2

MS2 and E. coli quantification 2

Plankton Enumeration 3

DAPI Cell Counts 3

Data analysis 3

Supplemental Figures **5**

Supplemental Tables **8**

Supplemental Methods

Glycerol stock preparation

Four American Type Culture Collection (ATCC) cultures were purchased for and used in this study, and they were propagated according to ATCC. MS2 (ATCC 15597-B1) was propagated in ATCC medium 271 with the recommended host (ATCC 15597) and recovered with Adam's Overlay method. E. coli strain HS(pF_{amp})R ("F_{amp}"; ATCC 700891) was propagated in ATCC medium 2553, which is tryptic soy broth (TSB) with 150 mg/L of streptomycin and ampicillin. E. coli K-12 (ATCC 29425) ('*E. coli*') was propagated in ATCC medium 3. All propagates were stored in cryovials with 20% glycerol at -80 °C until use ("glycerol stock"). In addition, *E. Faecalis* (ATCC 1934) was propagated from glycerol stocks in Luria Broth as a control for *E. coli* quantification.

E. coli, F_{amp}, and *E. faecalis* experimental preparation

Two days prior to T0, *E. coli*, F_{amp}, and *E. faecalis* stocks were thawed on ice, streaked onto plates (ATCC mediums 3, 2553, and 3 respectively), and incubated at 37°C overnight. Bacterial stocks did not undergo more than three freeze thaw cycles. One colony from each plate was inoculated into the corresponding ATCC broth, which was then incubated at 37°C overnight. On day T0, the *E. faecalis* overnight culture was placed at 4°C until use. For E. coli and F_{amp}, a loop of the overnight culture was used to inoculate TSB without antibiotics and TSB with 15 mg/L streptomycin and ampicillin respectively. The bacteria were brought to log-phase and then stored at 4°C until use (within 8 hours).

MS2 and E. coli quantification

MS2 was quantified using the double agar layer plaque assay in United States Environmental Protection Agency (USEPA) method 1602 with log-phase F_{amp} and tryptic soy broth (TSB) plates with 15 mg/L of streptomycin and ampicillin, as described previously (1). Briefly, each reactor sample was serially diluted in increments of 10 in autoclave-sterilized phosphate buffered saline (PBS; Thermo Fisher Scientific, Waltham, MA, USA). Five PBS dilutions were included per reactor at T0 and four to five PBS dilutions were included at T1 or T2. 200 µL of log-phase F_{amp} and 300 µL of each PBS dilution were combined in 4.5 mL of soft agar (0.7% agar), vortexed briefly, and then poured onto a hard agar plate (1.5% agar). The plates were incubated at 37°C for 16-24 hours, and then plaque-forming units (PFU) were enumerated for each plate. PFU were enumerated between 15 and 350 plaques, and plates above 350 PFU were considered too numerous to count.

E. coli was quantified following USEPA method 1603 on membrane Thermotolerant *E. coli* (mTEC) agar (Becton Dickenson, Franklin Lakes, NJ, USA), but the spread plate method was used for quantification. Each reactor sample was serially diluted in increments of 10 in autoclave-sterilized PBS. Four PBS dilutions were included per reactor at T0 and three to four PBS dilutions were included at T1 or T2. 20 µL of each dilution was plated onto mTEC agar plates. A glass spreader was flame-sterilized and allowed to cool before spreading the sample.

After spreading, the plates were incubated at room temperature for at least 20 minutes. The plates were placed upside down at 37°C for 22-24 hours, and then colony forming units (CFU) were enumerated for each plate. CFU were enumerated between 1 and 80 colonies, and plates above 80 CFU were considered too numerous to count.

Plankton Enumeration

For the raw water, plankton were concentrated onto 70 µm opening mesh, preserved with 70% ethanol, stained with 0.04% rose bengal, and enumerated by microscopy following previous methods (2). On the day of collection of each environmental water, ~12 to 28 L of raw water were poured over autoclave-sterilized, 70 µm opening, polyester mesh (Component Supply, Fort Meade, FL, USA). The mesh was rinsed with ~7.5 to 23 mL of 70% Biology Grade ethanol (Fisher, Waltham, MA, USA) to preserve plankton. The ethanol-preserved plankton were stored at 4°C until use within six months. At least 24 hours prior to microscopy, the plankton were stained with 0.04% rose bengal disodium salt (Fisher, Waltham, MA, USA). First, 5 mL of stained plankton were enumerated in a 60 x 15 mm plate using a Nikon SMZ800, and images were collected with an iPhone 11. If plankton were dense and difficult to differentiate, they were diluted with 70% ethanol 1:5 and the process was repeated until all identified plankton could be enumerated. Not all plankton could be identified, and counts from unidentified plankton were not included. Therefore, the plankton concentrations are conservative. The total identified plankton counts are the summation of all identified plankton with numerical counts (i.e., not including too numerous to count plankton) at each dilution.

DAPI Cell Counts

DAPI cell count was assessed with 4′,6-diamidino-2-phenylindole (DAPI) stain following methods described previously with slight modifications (3). Raw water samples were fixed with 2% paraformaldehyde on T0, and stored at -80°C until use within four months. Samples were thawed and vacuum-filtered through a 0.2 µm pore-size polycarbonate filter (MilliporeSigma, Burlington, MA, USA) with a 0.45 µm pore-size cellulose ester backing filter (MilliporeSigma, Burlington, MA, USA). The filters were rinsed with autoclave-sterilized PBS, a 1:1 mix of PBS and ethanol, and then ethanol. The polycarbonate filter was placed on a microscope slide, the filter was stained with 10 µL of Vectashied with DAPI (Fisher, Waltham, MA, USA), and a coverslip was placed over the filter. The slide was kept in the dark until imaging, which was completed on the same day as staining. The cells were imaged using an inverted epifluorescence microscope (Nikon, Minato City, Tokyo, Japan). Five to six images were collected per filter, and the cells were enumerated using ImageJ 1.53t (4).

Data analysis

Landry and Hassett (5) used a similar equation as equation 1 to characterize grazing in natural planktonic communities using terms “g” to represent the effect of gazers and “k” to represent the growth rate of the prey in the water. Herein, we applied a similar approach for seeded microorganisms, but renamed the coefficients “large particle coefficient” for the slope (instead of g) and “small particle coefficient” for the intercept (instead of k) because particles removed by

filter-sterilization include potential grazers but also other particles present in raw water that could affect seeded microorganism persistence.

Supplemental Figures

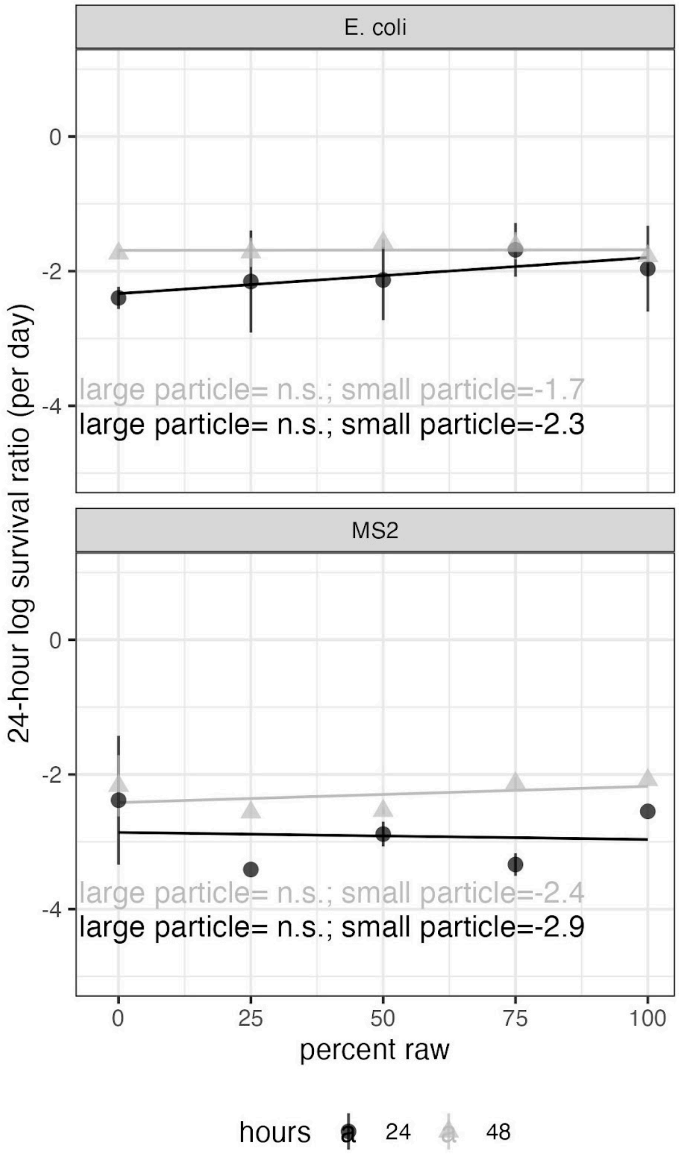

Figure S1: Water from San Gregorio State Beach was collected, and dilutions of raw water with filter-sterilized water (x-axis) were compared. For each dilution, the apparent rate of change (day^{-1}) was assessed after 24 (black) or 48 (gray) hours incubating in the dark at 15°C for *E. coli* (top) and MS2 (bottom). The error bars denote the standard deviation of duplicate reactors with water from the same grab sample. The coefficients were calculated using equation 1.

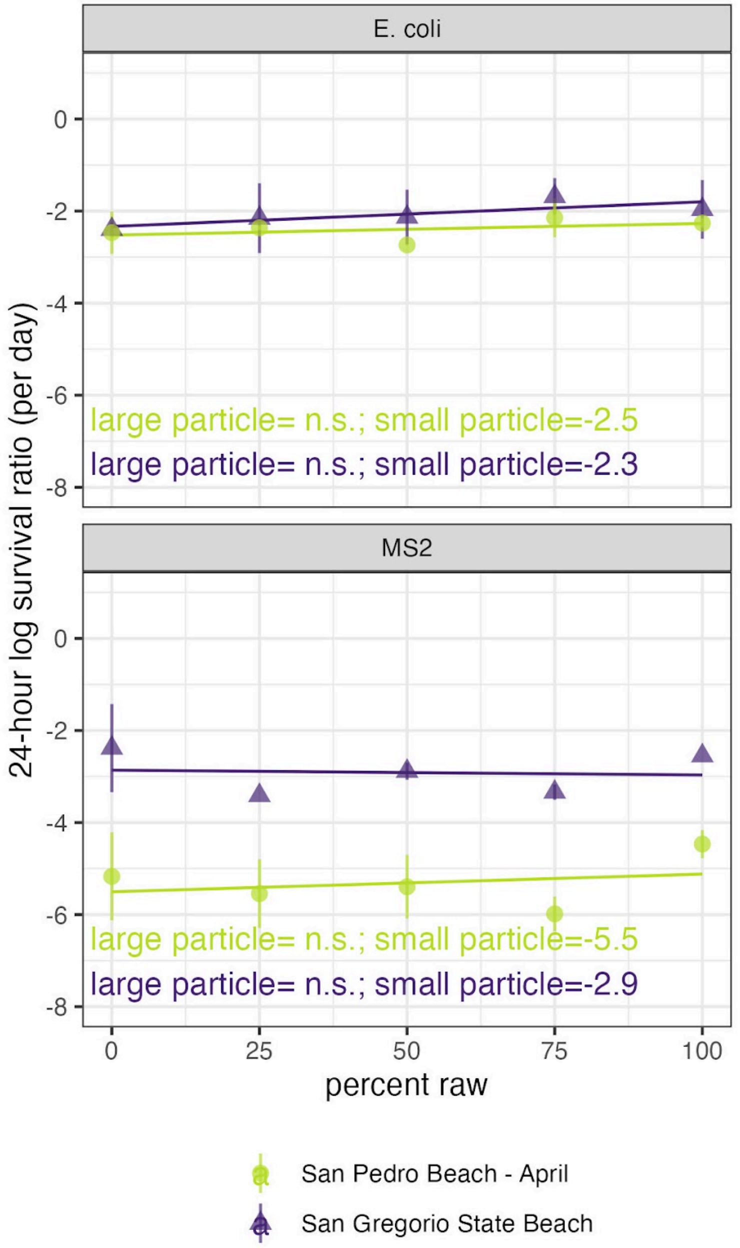

Figure S2: Water from San Gregorio State Beach and San Pedro Beach - April were compared (distinguished by color). Dilutions with filter-sterilized water consisted of 0, 25, 50, 75, and 100% raw water (x-axis), and the apparent rate of change (day^{-1}) was assessed after 24 hours incubating in the dark at 15°C for E. coli (top) and MS2 (bottom). The error bars denote the standard deviation of duplicate reactors with water from the same grab sample. The coefficients were calculated using equation 1.

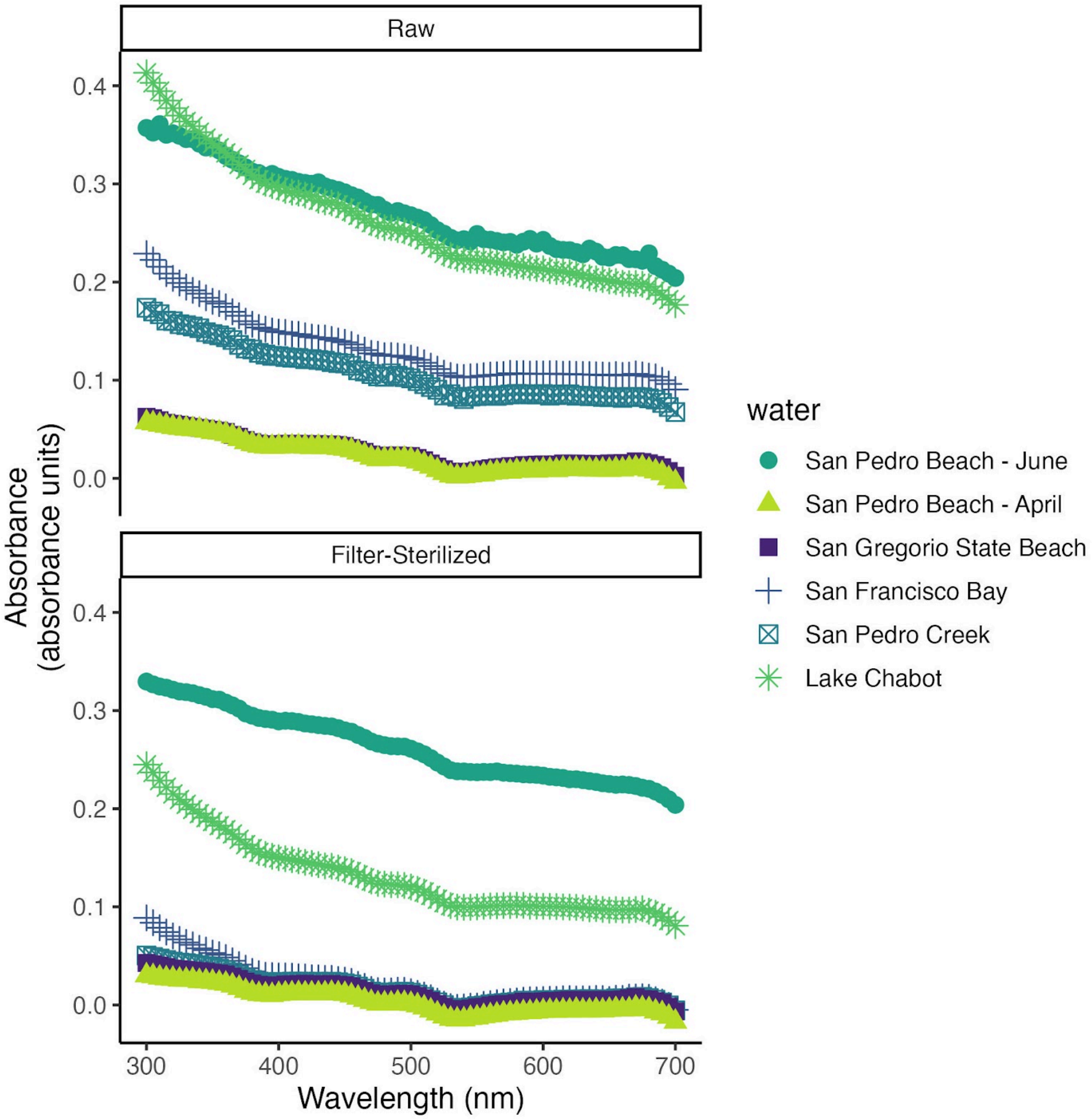

Figure S3: Absorbance of water samples from 250 to 700 nm in raw (top) or filter-sterilized (bottom) water for all environmental water samples included in this study (indicated by color and shape).

Supplemental Tables

Table S1: Large particle ("$\beta_{large\ particle}$") and small particle ("$\beta_{small\ particle}$") coefficients from equation 1 where the type of microorganism ("Type"), the environmental water tested ("Environmental water"), and the Incubation time ("Incubation time (h)") conditions are specified. The standard error and p-value of each coefficient are provided.

Type	Environmental water	Incubation time (h)	$\beta_{large\ particle}$	Standard error of $\beta_{large\ particle}$	p-value of $\beta_{large\ particle}$	$\beta_{small\ particle}$	Standard error of $\beta_{small\ particle}$	p-value of $\beta_{small\ particle}$
E. coli	San Gregorio State Beach	24	0.0054	0.0023	0.10	-2.3	0.14	<0.001
E. coli	San Gregorio State Beach	48	7.5×10^{-5}	0.0012	0.95	-1.7	0.074	<0.001
E. coli	San Pedro Beach - April	24	0.0025	0.0030	0.45	-2.5	0.18	<0.001
E. coli	San Pedro Creek	24	-0.0017	0.00068	0.052	-0.89	0.31	<0.05
E. coli	San Francisco Bay	24	-0.00014	0.00060	0.82	-1.1	0.25	<0.01
E. coli	Lake Chabot	24	-0.00046	0.00034	0.24	-0.33	0.15	0.076
E. coli	San Pedro Beach - June	24	0.0010	0.00038	<0.05	-3.3	0.17	<0.0001
MS2	San Gregorio State Beach	24	-0.0010	0.0067	0.90	-2.9	0.41	<0.01
MS2	San Gregorio State Beach	48	0.0024	0.0031	0.50	-2.4	0.19	<0.01
MS2	San Pedro Beach - April	24	0.0039	0.0078	0.66	-5.5	0.48	<0.01
MS2	San Pedro Creek	24	0.00034	0.00045	0.49	-0.47	0.20	0.071
MS2	San Francisco Bay	24	0.0013	0.0011	0.27	-3.2	0.44	<0.001
MS2	Lake Chabot	24	0.0012	0.00040	<0.05	-1.5	0.17	<0.001
MS2	San Pedro Beach - June	24	0.0020	0.00088	0.072	-4.5	0.39	<0.0001

Table S2: Multiple regression coefficients for equation 2, which was used to assess the effect of incubation time on the large and small particle decay coefficients for MS2. The residual standard error was 0.41 on 3 and 6 degrees of freedom (adjusted R2 = 0.24; p =0.22).

term	estimate	standard error	statistic	p value
β_0	-3.3	0.71	-4.6	0.0036
β_1	-0.0045	0.012	-0.38	0.71
β_2	0.44	0.45	0.98	0.36
β_{12}	0.0035	0.0074	0.47	0.66

Table S3: Multiple regression coefficients for equation 2, which was used to assess the effect of incubation time on the large and small particle decay coefficients for *E. coli*. The residual standard error was 0.15 on 3 and 6 degrees of freedom (adjusted R2 = 0.71; p <0.05).

term	estimate	standard error	statistic	p value
β_0	-3.0	0.25	-12	<0.0001
β_1	0.011	0.0041	2.6	0.042
β_2	0.64	0.16	4.0	0.0069
β_{12}	-0.0053	0.0026	-2.0	0.089

Table S4: Multiple regression coefficients for equation 3, which was used to assess whether large and small particle decay coefficients for E. coli were different between environmental waters tested. The residual standard error was 0.21 on 3 and 6 degrees of freedom (adjusted R2 = 0.48; p = 0.080).

term	estimate	standard error	statistic	p value
β_0	-2.3	0.16	-14	<0.0001
β_1	0.0054	0.0026	2.0	0.089
β_2	-0.19	0.23	-0.82	0.44
β_{12}	-0.0028	0.0037	-0.75	0.48

Table S5: Multiple regression coefficients for equation 3, which was used to assess whether large and small particle decay coefficients for MS2 were different between environmental waters tested. The residual standard error was 0.58 on 3 and 6 degrees of freedom (adjusted R2 = 0.82; p <0.01).

term	estimate	standard error	statistic	p value
β_0	-2.9	0.45	-6.4	<0.001
β_1	-0.0010	0.0073	-0.14	0.89
β_2	-2.6	0.63	-4.2	<0.01
β_{12}	0.0049	0.010	0.47	0.65

Table S6: Multiple regression coefficients for equation 4, which was used to assess whether large and small particle decay coefficients for *E. coli* were different between environmental waters tested. The residual standard error was 0.48 on 7 and 20 degrees of freedom (adjusted R2 =0.81 X; p< 0.0001).

term	estimate	standard error	statistic	p value
β_0	-0.33	0.23	-1.5	0.16
β_1	-0.00046	0.00053	-0.87	0.40
β_2	-0.75	0.32	-2.4	<0.05
β_3	-0.56	0.32	-1.7	0.096
β_4	-2.9	0.32	-9.2	<0.0001
β_{12}	0.00031	0.00075	0.41	0.68
β_{13}	-0.0013	0.00073	-1.7	0.097
β_{14}	0.0015	0.00073	2.1	0.054

Table S7: Multiple regression coefficients for equation 4, which was used to assess whether large and small particle decay coefficients for MS2 were different between environmental waters tested. The residual standard error was 0.69 on 7 and 20 degrees of freedom (adjusted R2 =0.82 X; p< 0.0001).

term	estimate	standard error	statistic	p value
β_0	-1.5	0.32	-4.5	<0.001
β_1	0.0012	0.00075	1.5	0.14
β_2	-1.8	0.46	-3.9	<0.01
β_3	0.99	0.46	2.2	<0.05
β_4	-3.1	0.46	-6.7	<0.0001
β_{12}	0.00016	0.0011	0.15	0.88
β_{13}	-0.00082	0.0010	-0.79	0.44
β_{14}	0.00084	0.0010	0.80	0.43

Table S8: Physicochemical water quality and ATP data for raw ("R") and filter-sterilized ("F") environmental water samples included in this study. Data that were not collected are denoted "-." Collection temperature and salinity were assessed onsite during sample collection.

Water	Matrix	Collection temperature (°C)	Salinity (ppt)	pH	Turbidity (NTU)
San Gregorio State Beach	R	11.3	24.3	7.86	5.4
San Gregorio State Beach	F	-	-	7.93	< 1
San Pedro Beach - April	R	11.2	32.1	7.78	8.1
San Pedro Beach - April	F	-	-	7.84	< 1
San Pedro Creek	R	12.9	0.2	8.02	5.6
San Pedro Creek	F	-	-	8.03	< 1
San Francisco Bay	R	17.7	15	7.99	14.8
San Francisco Bay	F	-	-	8.03	< 1
Lake Chabot	R	20.6	0.2	8.05	6.1
Lake Chabot	F	-	-	8.08	2.3
San Pedro Beach - June	R	14.2	32.7	8.00	5.6
San Pedro Beach - June	F	-	-	8.03	1.6
